# Neoalbaconol induces cell death through necroptosis by regulating RIPK-dependent autocrine TNFα and ROS production

**DOI:** 10.18632/oncotarget.3038

**Published:** 2014-12-03

**Authors:** Xinfang Yu, Qipan Deng, Wei Li, Lanbo Xiao, Xiangjian Luo, Xiaolan Liu, Lifang Yang, Songling Peng, Zhihui Ding, Tao Feng, Jian Zhou, Jia Fan, Ann M. Bode, Zigang Dong, Jikai Liu, Ya Cao

**Affiliations:** ^1^ Cancer Research Institute, Xiangya School of Medicine, Central South University, Hunan, China; ^2^ Key Laboratory of Chinese Ministry of Education, Central South University, Hunan, China; ^3^ Key Laboratory of Carcinogenesis of Chinese Ministry of Public Health, Central South University, Hunan, China; ^4^ State Key Laboratory of Phytochemistry and Plant Resources in West China, Kunming Institute of Botany, Chinese Academy of Sciences, Yunnan, China; ^5^ The Hormel Institute, University of Minnesota, Austin, Minnesota, USA; ^6^ Liver Cancer Institute, Liver Surgery Department, Zhongshan Hospital

**Keywords:** necoalbaconol, necroptosis, RIPK, TNFα, NF-κB signaling pathway, ROS

## Abstract

Necroptosis/regulated necrosis is a caspase-independent, but receptor interacting protein kinase (RIPK)-dependent form of cell death. In previous studies, neoalbaconol (NA), a constituent extracted from *Albatrellus confluens*, was demonstrated to induce necroptosis in some cancer cell lines. The molecular mechanism of NA-induced necroptosis is described in this research study. We determined that NA-induced cell death is partly dependent on tumor necrosis factor α (TNFα) feed-forward signaling. More importantly, NA abolished the ubiquitination of RIPK1 by down-regulating E3 ubiquitin ligases, cellular inhibitors of apoptosis protein 1/2 (cIAP1/2) and TNFα receptor-associated factors (TRAFs). The suppression of RIPK1 ubiquitination induced the activation of the non-canonical nuclear factor-κB (NF-κB) pathway and stimulated the transcription of TNFα. Moreover, we also found that NA caused RIPK3-mediated reactive oxygen species (ROS) production and contribution to cell death. Taken together, these results suggested that two distinct mechanisms are involved in NA-induced necroptosis and include RIPK1/NF-κB-dependent expression of TNFα and RIPK3-dependent generation of ROS.

## INTRODUCTION

Regulated cell death, a physiologic process for elimination of damaged cells, is critically important in normal development and disease pathogenesis. Multiple mechanisms of regulated cell death have been identified that function in distinct manners: apoptosis, autophagic cell death, and necrosis. Necroptosis is a form of regulated necrosis that is RIPK1/3-dependent under apoptotic deficient conditions [1]. Several types of stimuli, including ligands of death receptors (such as Fas, TRAIL and TNFα), virus infection, and anticancer agents, can initiate necroptosis [2]. Resistance to cell death is a hallmark of human cancer. Because necroptosis can act as an alternative cell death pathway when apoptosis is compromised, it might be exploited as a novel anticancer strategy [3]. Research results showed that a growing list of anticancer agents, such as etoposide [4], Smac mimetic [5, 6] and shikonin [7], can initiate necroptosis to kill cancer cells. These findings raised the possibility that necroptosis might be considered as an alternative choice for cancer treatment.

TNFα, a major mediator of inflammation, cell survival and cell death depending on cellular context, is the most extensively studied initiator of necroptosis. Previous studies showed that zVAD-fmk and IAP antagonist could induce necroptosis in cancer cells that was dependent on autocrine TNFα production [8, 9]. By binding with TNFR1, TNFα triggers the formation of the TNFR1 signaling complex by recruiting several effectors such as TNFR-associated death domain (TRADD), RIPK1, cIAP1/2, and TRAFs [10, 11]. In particular, ubiquitination, de-ubiquitination and the interaction of RIPK1 with different adaptor proteins control cell fate [10, 11]. RIPK1 is ubiquitinated by cIAP1/2 and other E3 ubiquitin ligases [12]. Interestingly, certain anticancer agents, such as etoposide, can suppress cIAP1 expression, thereby inducing formation of a complex called the ripoptosome, which comprises RIPK1, FADD, RIP3 and caspase 8, resulting in necroptosis [4, 13]. Lys63-polyubiquitylated RIPK1 binds to the transforming growth factor-β-activated kinase 1 (TAK1)/TAK1-binding protein 2 (TAB2)/TAB3 complex, leading to the activation of NF-κB and transactivation of cytoprotective genes to facilitate cell survival [10]. Under certain conditions, the RIPK1 Lys63-de-ubiquitination regulated by deubiquitinase cylindromatosis (CYLD) and A20 switches its function from survival promotion to cell death [14]. The execution of necroptosis requires RIPK1 and RIPK3 kinase activity in the necrosome [11]. Activated RIPK3 binding with mixed lineage kinase-domain like (MLKL) results in ROS production and necroptotic cell death [15].

Two different NF-κB pathways, referred to as the canonical and non-canonical pathways, play critical roles in the regulation of diverse biological processes [16]. The activation of the canonical NF-κB pathway is mediated by the phosphorylation of IκBα by IKK components (IKKα, IKKβ and IKKγ, primarily IKKβ) and subsequent degradation of IκBα, which results in the nuclear translocation of the p65-p50 heterodimer, leading to gene transcription [17]. Unlike IκB degradation, the activation of the non-canonical NF-κB pathway is dependent on IKKα and its activator, NF-κB inducing kinase (NIK), but independent of IKKβ and IKKγ [17]. Accumulated NIK phosphorylates and activates inhibitor of IKKα, which in turn phosphorylates p100 [18]. Upon phosphorylation by IKKα, p100 is processed in the proteasome to generate p52; the p52-RelB dimer enters the nucleus and activates NF-κB-responsive genes to transcription [19]. Studies showed that loss of cIAPs facilitates the stabilization of NIK, promoting IKKα/IKKα activation and NF-κB2 p100 processing to p52 [18, 20]. The non-canonical NF-κB-driven pathway up-regulates specific target genes, including *TNF-α* that is required for Smac mimetic-induced cell death [20, 21]. In particular, cIAP1/2 is required for stimulus-dependent activation of the canonical pathway and for constitutive suppression of the non-canonical NF-κB pathway. Under non-stimulated conditions, non-canonical NF-κB signaling is suppressed by cIAP-mediated degradation of NIK [18, 20, 21]. cIAP-mediated degradation of NIK also requires TRAF2 and TRAF3, which function as adaptor proteins recruiting NIK to cIAPs. Loss of TRAF2, TRAF3 or cIAPs prevents NIK turnover and results in the accumulation of NIK protein levels and in the stimulation of non-canonical NF-κB signaling [22]. For the canonical NF-κB pathway, stimulation with TNFα leads to recruitment of cIAPs through TRAF2 to the plasma membrane-bound TNFR1 signaling complex, resulting in the activation of the canonical NF-κB pathway [23].

Our group previously reported that neoalbaconol (NA), a novel small-molecular compound isolated from the fungus, *Albatrellus confluens,* could activate autophagy and cause apoptotic and necroptotic cell death through an independent pathway [24]. Necroptosis was markedly induced, which was confirmed by the presence of necrotic morphology, and rescued by the necroptosis inhibitor necrostatin-1 (Nec-1) [24]. Here, we report that NA-induced cell death is dependent on TNFα feed-forward signaling. Furthermore, ROS production through RIPK3 also contributed to cell death in NA-treated cells. These findings provide novel insights into the molecular mechanisms of NA-induced necroptosis of cancer cells and suggest that NA may be a potential therapeutic agent in the treatment of cancer.

## RESULTS

### Autocrine production of TNFα correlates with RIPK-dependent necroptosis in response to NA

**Figure 1:**
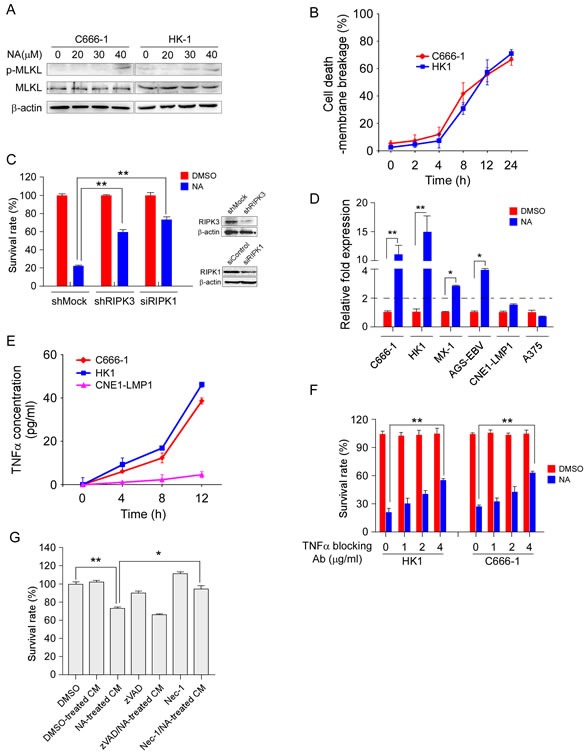
NA promotes autocrine production of TNFα and is required for necroptosis A. MLKL phosphorylation was detected using an MLKL phosphor-specific antibody. C666-1 and HK1 cells were treated with NA for 8 h and then harvested. Whole-cell lysates were subjected to SDS-PAGE followed by Western blot analysis. β-Actin is shown as a loading control. B. The number of dead cells was determined by measuring membrane integrity. C666-1 and HK1 cells were treated with NA and harvested at the indicated time points and membrane integrity was determined by trypan blue staining. C. RIPK1 and RIPK3 expression was knocked down in C666-1 cells, and then cells were treated with NA. Cell viability was estimated by MTS assay. D. NA treatment promotes TNFα transcription. Cells were treated with NA (40 μM) for 8 h and the *TNFα* mRNA level was determined by quantitative real time-PCR. E. NA triggers autocrine production of TNFα. Cells were treated as the indicated time points. Supernatant fractions from control and NA-stimulated cells were removed at the indicated time points and the secreted TNFα level was measured by ELISA. F. Autocrine signaling is required for NA-induced cell death. C666-1 cells were pre-treated (1 h) with neutralizing antibodies (1-4 μg/mL) against TNFα prior to treatment with 40 μM NA. Cell viability was estimated by MTS assay. G. Soluble factors mediate the anti-proliferation effect of NA. Cells were treated for 4 h with NA, washed with PBS 3 times, and fresh medium was added and cells incubated for another 6 h. At that time, the medium was collected as conditioned medium. Each graphical representation indicates the means ± S.D. of at least three independent testing conditions. *p<0.05. **p<0.001.

In previous study, we found that NA can induce apoptotic and necroptotic cancer cell death through an independent pathway. Phosphorylation of Thr357 and Ser358 of MLKL is a specific cellular marker of necroptosis [15, 25]. To detect necroptosis in NA-treated cells, an antibody against the phosphorylation of Thr357/Ser358 of human MLKL was used by Western blot analysis. The phosphorylation of MLKL was up-regulated in NA-treated human nasopharyngeal carcinoma C666-1 and HK1 cells (Figure 1A). Necrotic cell death has also been marked by the loss of cytoplasmic membrane integrity, which can be measured by trypan blue staining. C666-1 and HK1 cells were treated with NA and then cell membrane integrity was analyzed at different time points. The loss of cytoplasmic membrane integrity started 4 h after treatment and continued with linear kinetics up to 12 h (Figure 1B). RIPKs are well-established as having a critical function in necroptosis. Knockdown of RIPK1 and RIPK3 reduced cell death induced by NA. These data suggest that NA induced both RIPK1- and RIPK3-dependent necroptotic cell death (Figure 1C).

Autocrine production of TNFα has been recognized as a critical signal for the induction of necroptosis [8, 9]. In the previous study, several cancer cell lines were screened with NA and some were killed, whereas others showed no reduction in viability. Here, a panel of cell lines was tested to consider the possibility that TNFα is involved in NA-induced cell death. After 8 h of NA treatment, cell lines (C666-1, HK1, MX-1, AGS-EBV) that showed sensitivity to NA showed a 3- to 15-fold induction of TNFα compared to untreated cells, whereas ‘resistant’ cells (A375, CNE1-LMP1) showed less than a 2-fold induction compared to control cells (Figure 1D). Exposure to NA also elevated the transcription of TNFα in L929 cells (Supplementary Figure 1A). Elisa analysis results demonstrated that after NA treatment cell lines sensitive to NA (C666-1, HK1) were secreting TNFα into the cell culture medium, whereas low levels of TNFα were present in medium of the resistant cell line (CNE1-LMP1) (Figure 1E). We further determined whether NA-induced secretion of TNFα contributes to cell death. Cells were pretreated with a neutralizing TNFα antibody and then treated with NA. The TNFα neutralizing antibody partially and dose-dependently rescued cells from death (Figure 1F). The conditioned medium from C666-1 cells treated with NA decreased cell viability compared with the conditioned medium from DMSO-treated cells (Figure 1G). More importantly, the C666-1 conditioned medium-mediated death effect could be rescued by Nec-1, but not zVAD (Figure 1G). zVAD could not up-regulate the *TNFα* mRNA level in C666-1 and HK1 cells (Supplementary Figure 1B), so, there may be a unique mechanism of NA-induced TNFα upregulation. Although, we cannot rule out the possibility that other factors might have a role in NA-induced cell death, our research clearly showed that autocrine TNFα plays a crucial role in NA-induced necroptotic cell death.

### NA induces degradation of cIAP1/2 in a proteasomal-dependent manner

**Figure 2:**
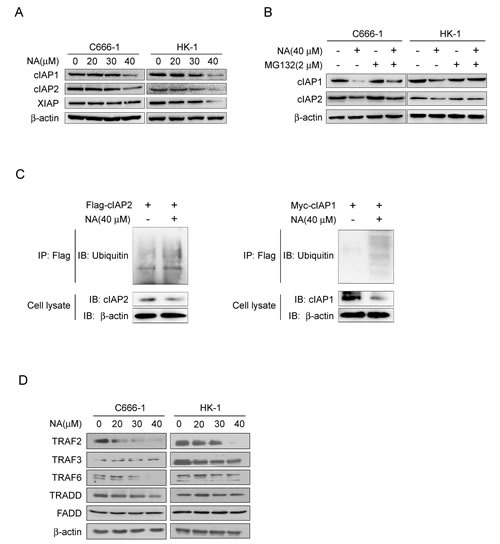
NA induces auto-ubiquitination and proteasomal degradation of cIAP1/2 A. NA causes loss of cIAP1 and cIAP2. C666-1 and HK1 cells were treated with NA at the indicated dose for 8 h and cell lysates were examined by Western blotting using antibodies against cIAP1 and cIAP2. β-Actin is shown as a loading control. B. NA-induced degradation of cIAP1 and cIAP2 is dependent on proteasomal machinery. C666-1 and HK1 cells were treated with NA (40 μM) for 1 h in the absence or presence of proteasome inhibitor (2 mM MG132) and cell lysates were examined by Western blotting using antibodies against cIAP1 and cIAP2. C. NA stimulates auto-ubiquitination of c-IAP1 and cIAP2. C666-1 cells were transfected with the *cIAP1-myc* or *cIAP2-flag* plasmids for 48 h, then treated or not treated with 40 μM NA for 8 h. cIAP1 and cIAP2 were immunoprecipitated and immunoblotted. β-Actin served as a loading control. D. C666-1 and HK1 cells were exposed to increasing concentrations of NA for 8 h, then lysed and immunoblotted. β-Actin served as a loading control.

Cells exposed to IAP antagonists can induce cIAP1/2 degradation, leading to RIPK1 de-ubquitination and autocrine TNFα to trigger either apoptotic or necroptotic cell death [12, 26, 27]. To determine whether cIAP1/2 can be affected by NA, we examined the cIAP1/2 protein level in cells incubated with or without NA. Results demonstrated that NA caused loss of cIAP1 and cIAP2 proteins and the XIAP protein level was also reduced at the same time in these cell lines (Figure 2A). IAP antagonists can induce loss of cIAP1 and cIAP2 by inducing their auto-ubiquitination and proteosomal degradation. To address possible mechanisms of NA-induced loss of cIAP1 and 2, C666-1 and HK1 cells were treated with NA in the absence or presence of the proteasome inhibitor, MG132. The presence of MG132 efficiently blocked the NA-dependent decreases in c-IAP1/2 protein levels (Figure 2B). NA exposure leading to enhancement in cIAP1 and cIAP2 ubiquitination further indicated that loss of cIAP1 and cIAP2 involves their auto-ubiquitination (Figure 2C). TRAF2, an E3 ubiquitin ligase, was originally identified as a cIAP1/2-binding protein mediating IAP antagonist-induced degradation of cellular cIAP1 [28-30]. Knockdown of TRAF2 in L929 cells activated RIPK1-dependent TNFα production and induced necroptosis. The protein levels of TRAF2 and TRAF6 were reduced in cancer cell lines treated with NA, but not TRAF3, TRADD, and FADD (Figure 2D). Collectively, these results demonstrate that NA triggers auto-ubiquitination and subsequent proteasomal degradation of cIAP1 and cIAP2.

### NA abolishes RIPK1 ubiquitination and activates the non-canonical NF-κB signaling pathway

**Figure 3:**
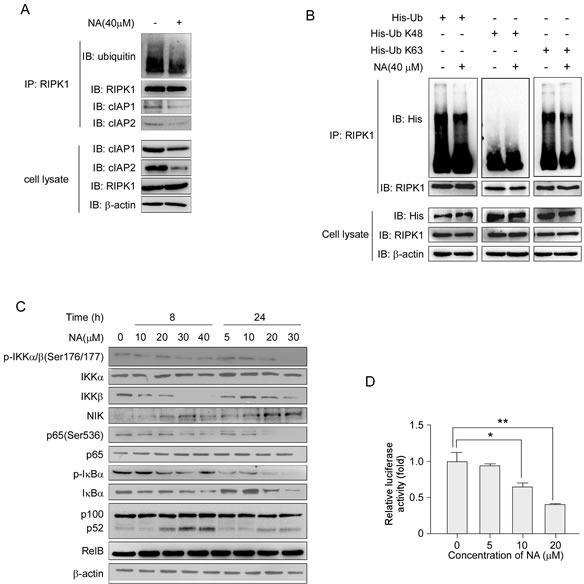
NA treatment abolishes RIPK1 ubiquitination and activates the NF-κB non-canonical pathway A. C666-1 cells were treated or not treated with 40 μM NA for 8 h, and RIPK1 was immunoprecipitated and immunoblotted. β-Actin served as a loading control. B. Cells were transfected with the His-Ub (wt, K48, K63) plasmids for 48 h, then treated or not treated with 40 μM NA for 8 h. RIPK1 was immunoprecipitated and immunoblotted. β-Actin served as a loading control. C. The effect of increasing doses of NA (0-40 μM) treatment for 8 or 24 h on the expression level of IKKα, IKKβ and NIK and downstream molecules, p65, p100, p52 and IκBα was analyzed by immunoblotting. β-Actin served as a loading control. D. NA inhibits *NF-κB reporter* gene expression. C666-1 cells were transiently transfected with an NF-κB-containing plasmid for 24 h. After transfection, cells were treated with the indicated concentrations of NA for 24 h. Gene expression was assayed by measuring luciferase activity.

Previous studies showed that triggering proteasomal degradation of cIAP1/2 could activate RIPK1 de-ubiquitination to induce cell death [12, 31]. Having established that NA could down-regulate cIAP1 and cIAP2, we examined the RIPK1 ubquitination level in cells treated or not treated with NA. Results showed that NA exposure led to a reduction in RIPK1 ubiquitination in C666-1 cells (Figure 3A). In cells treated with NA, the endogenous levels of cIAP1 and cIAP2 were decreased by co-immunoprecipitation with an anti-RIPK1 antibody (Figure 3A). Different types of ubiquitination modifications of RIPK1 can lead to different functions. To determine which ubiquitination modification of RIPK1 was affected by NA, *Wt-Ub, His-UbK48* or *His-UbK63* plasmids were introduced into C666-1 cells. NA treatment reduced the ubiquitination of the K63-linked modification compared with the control, whereas the ubiquitination of the K48-linked modification was not affected (Figure 3B).

Either cIAP1 or cIAP2 is required for RIPK1 polyubiquitination and NF-κB activation upon TNFα treatment [32-34]. Having established that RIPK1 ubiquitination is reduced, we next investigated the signaling pathways that are regulated by RIPK1, including the canonical and non-canonical NF-κB pathways. Western blot analysis showed that treatment with NA reduced IKKβ and inhibited phosphorylation of p65 compared to untreated cells, but IKKα was not changed (Figure 3C). Previous reports showed that IAP antagonist-induced proteasomal degradation of cIAP1/2 would eliminate the candidate E3 ligases responsible for proteasomal degradation of NIK, thus allowing NIK to accumulate and trigger the non-canonical NF-κB pathway [20]. Here, we found that NIK protein levels increased and were accompanied by the processing of p100 to p52 following NA treatment (Figure 3C). The Rel-B protein level was not changed after NA treatment (Figure 3C). The translocation of p65 to the nucleus is preceded by the proteolytic degradation of IκBα. NA at a high dose has a slight effect on IκBα degradation up to 24 h (Figure 3C). Whether NA could suppress constitutive NF-κB/p65 activation was also examined. Reporter gene data suggested that constitutive NF-κB/p65 activation was suppressed by NA at various concentrations (Figure 3D). Taken together, these findings indicate that NA reduced the K63 ubiquitination of RIPK1, inhibited the canonical NF-κB pathway and activated non-canonical NF-κB pathway, respectively.

### The non-canonical NF-κB pathway has a key role in NA-induced TNFα production and cell death

**Figure 4:**
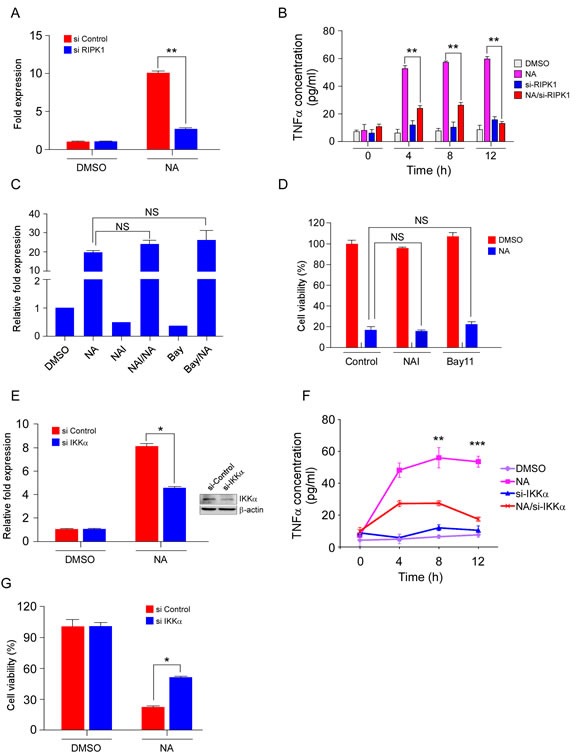
The RIPK/NF-κB pathway mediates NA-induced TNFα production and cell death.cA C666-1 cells transfected for 48 h with siRNA targeting RIPK1 or an empty vector control were treated or not treated with 40 μM NA for 8 h and relative levels of the TNFα transcript were determined and compared with β-actin and the fold change was calculated by comparing with DMSO-treated cells. B. C666-1 cells transfected for 48 h with siRNA targeting RIPK1 or an empty vector control were treated or not treated with 40 μM NA and harvested at the indicated time points. The presence of TNFα in conditioned cell culture media was measured by Elisa assay. C. The effect of NAI and Bay117082 (Bay) on NA-induced TNFα transcription. Cells were pre-treated with NAI (40 μM) and Bay117082 (Bay) (5 μM) for 1 h, and then treated or not treated with NA (40 μM). *TNFα* mRNA level was analyzed by quantitative-real time-PCR. D. Cells were pre-treated with NAI (40 μM) and Bay117082 (Bay) (5 μM) for 1 h, and then treated or not treated with NA (40 μM). Cell viability was estimated by MTS assay. E. IKKα in C666-1 cells was knocked down with siRNA, and then cells were treated with NA. *TNFα* mRNA level was analyzed by quantitative-real time-PCR. F. C666-1 cells transfected for 48 h with siRNA targeting IKKα or an empty vector control were treated or not treated with 40 μM NA and harvested at the indicated time points. The presence of TNFα in conditioned cell culture media was measured by Elisa assay. G. IKKα in C666-1 cells was knocked down with siRNA, and then cells were treated with different doses of NA. Viability of C666-1 cells was analyzed by MTS assay. Data are shown as means ± S.D. of values from three independent experiments. *p<0.05. **p<0.001. ***p<0.0001

Researchers reported that RIPK1 and JNKs mediated TNFα production in response to zVAD-fmk and IAP antagonists [8, 9]. Thus, the roles of RIPK1 and JNKs were analyzed to further investigate the molecular mechanisms of TNFα production in NA-treated cells. To determine how TNFα production is activated, we used real-time PCR to measure *TNFα* mRNA levels after NA treatment. Stimulation with NA increased *TNFα* mRNA expression, and knockdown of RIPK1, blocked the increase in *TNFα* mRNA level (Figure 4A). Thus, RIPK1 activates the transcription of TNFα after NA treatment. In a previous study, we found that NA treatment could induce JNKs activation[24]. SP600125 (SP), a small molecular inhibitor of JNKs, was used to verify the possibility that JNKs activation may trigger autocrine production of TNFα. SP treatment could not reduce *TNFα* transcription level induced by NA (Supplementary Figure 2A). Furthermore, results showed that knockdown of RIPK1 inhibited NA-induced TNFα secretion (Figure 4B), demonstrating that RIPK1 is required for TNFα production. In addition, inhibition of JNKs by SP treatment failed to block NA-induced TNFα production (Supplementary Figure 2B). Although JNKs were phosphorylated and activated by NA treatment, NA-induced TNFα secretion occurred independently of JNKs activation. JNKs activation might be a side effect of NA stimulation. Therefore, these results implied that RIPK1, but not JNKs, contributes to NA-induced TNFα production.

Activation of the NF-κB pathway is usually thought to promote cell survival. However, certain situations also exist in which activation of NF-κB is pro-apoptotic and might lead to the production of TNFα and FasL [35]. Both the canonical and non-canonical NF-κB pathways have been reported to participate in autocrine production of TNFα in cancer cells [20, 21, 36]. We examined the role of canonical and non-canonical NF-κB pathways in TNFα production after treatment with NA. The NF-κB activation inhibitor (NAI) and BAY11-7082 (BAY11), which are small molecular inhibitors of the canonical NF-κB pathway, did not affect NA-induced transcription of TNFα (Figure 4C). In addition, NAI and BAY11 could not rescue the cells from death (Figure 4D). siRNA silencing of IKKα, a key molecule in the non-canonical NF-κB pathway, down-regulated the transcription and secretion levels of TNFα (Figure 4E, F). More importantly, NA-induced TNFα-dependent necrotic cell death was inhibited by silencing IKKα (Figure 4G). Consistent with the inhibition of the canonical pathway and promotion of the non-canonical pathway, pro-survival and anti-apoptotic genes regulated by canonical NF-κB were decreased with NA treatment, whereas the IL-1α and IL-8 were up-regulated (Supplementary Figure 3). Thus, these results indicated that the non-canonical NF-κB pathway has a key role in NA-induced TNFα production and cell death, and after NA treatment, the secretome may also include other factors that promote cell death, such as ILs.

### NA-mediated ROS generation contributes to cell death

**Figure 5:**
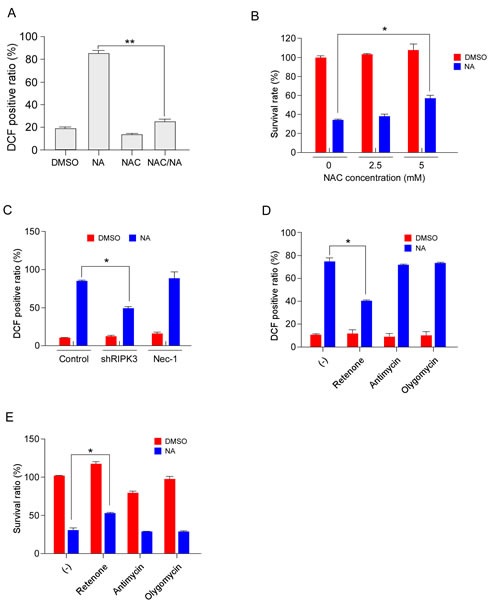
NA-mediated ROS generation contributes to cancer cell death A. C666-1 cells were pre-treated with the ROS scavenger, NAC (5 mM), and then treated or not treated with 40 μM NA. ROS were measured using the dye DCF at 12 h after stimulation. B. C666-1 cells were pre-treated with NAC for 1 h, and then treated or not treated with NA (40 μM) for 12 h. Cell viability was estimated by MTS assay. C. C666-1 cells transfected for 48 h with shRNA targeting RIPK3 or an empty vector control were treated or not treated with 40 μM NA. ROS were measured using the dye DCF at 12 h after stimulation. D-E. Cells were treated or not treated with neoalbaconol (40 μM) in the presence of rotenone (1 μM), antimycin A (40 μM), or oligomycin (1 μM). (d) The ROS levels were measured 12 h after stimulation. (e) The cell viability was estimated by MTS assay at 24 h after stimulation. Data are shown as means ± S.D. of values obtained from three independent experiments. *p<0.05. **p<0.001.

Classically, execution of necroptosis has been thought to involve ROS generation and mitochondrial dysfunction [37, 38]. Here, we investigated whether ROS participate in NA-induced cell death. Results showed that the amount of ROS was markedly elevated by NA treatment (Figure 5A). Pretreatment of C666-1 cells with the ROS scavenger N-acetyl-L-cysteine (NAC) decreased ROS production (Figure 5A). NAC administration also attenuated NA-induced cell death (Figure 5B), indicating that ROS production contributed to NA-induced cell death. RIPK3 is key regulator in energy metabolism-associated ROS production, which partially accounts for RIPK3′s ability to promote necrosis [39]. Knocking down RIPK3 reduced ROS production in NA-treated cells, whereas treatment with the RIPK1 inhibitor Nec-1 had no effect on ROS production induced by NA (Figure 5C). Knocking down of IKKα did not change ROS production in NA-treated cells (Supplementary Figure 4A). This suggested that ROS production independent from activation of the non-canonical NF-κB pathway. Mitochondrial complex I and complex III are major ROS-producing sites in the electron transport chain (ETC). Several inhibitors of energy metabolism were used to confirm that the source of ROS originates from the mitochondria. The mitochondrial complex I inhibitor, rotenone, reduced the ROS production in NA-treated cells; but the complex III inhibitor, antimycin, and the ATPase inhibitor, olygomycin, had no effect (Figure 5D). Furthermore, rotenone attenuated NA-induced cell death (Figure 5E). Previous studies showed that besides mitochondria, the activation of phospholipases/lipoxygenases (PLA2/LOX), also contributes to ROS production, and consequently to necrotic cell death [40]. The LOX inhibitor (AA861) and PLA2 inhibitor (bromoenol lactone, BEL) were used to exclude this possibility. AA861 and BEL had no effect on ROS production in NA-exposed cells (Supplementary Figure 4B) and NA-induced cell death was not changed by AA861 and BEL (Supplementary Figure 4B). Collectively, these data suggested that the mitochondrial complex I is the main site for ROS generation and main contributor to cell death in NA-treated cells.

## DISCUSSION

**Figure 6:**
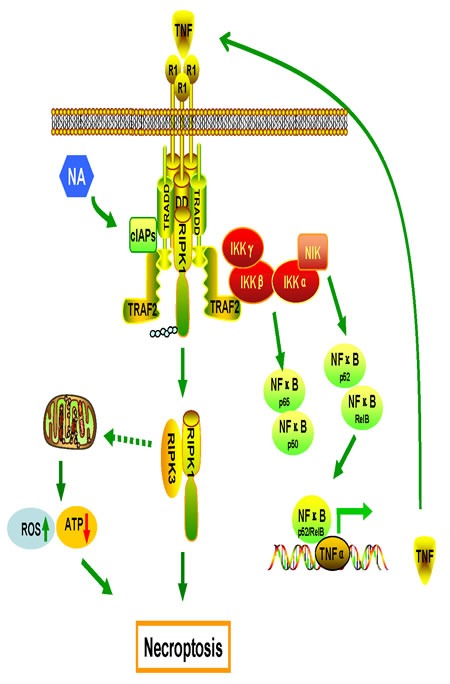
Proposed model of the mechanisms of NA-induced cell death Production of TNFα by the RIPK/NF-κB signaling pathway and ROS from mitochondria are required for necroptosis induced by NA.

In this study, we discovered that NA triggers necroptosis by promoting autocrine secretion of TNFα through the regulation of the RIPK/NF-κB signaling pathway and RIPK3-dependent ROS production. NA induces the proteasomal degradation of cIAPs that leads to the de-ubiquitination of RIPK1 which activating necroptosome to initiate necroptosis; meanwhile, loss of cIAPs facilitates the stabilization of NIK, promoting IKKα/IKKα activation and the processing of p100 to p52 to activate non-canonical NF-κB pathway. The non-canonical NF-κB-driven pathway up-regulates specific target genes, including *TNFα* that is required for NA-induced cell death. Surprisingly, NA reduced the phosphorylation of p65 to inhibit the canonical NF-κB pathway, providing the insight that NA not only activate the pro-cell death signal of NF-κB, but also inhibits the pro-survival signal of this pathway. Taken together, data from this study suggest that NA is capable of inducing necroptosis by these mechanisms (illustrated in Figure 6).

TNFα, a pleiotropic ligand of TNFR1/2, can signal both cell survival and cell death based on the downstream protein complexes constitutes. The ubiquitination, de-ubiquitination, and the interaction of RIPK1 with a class of ubiquitin receptors control cell fate [41]. TNFα is produced in two ways. First, TNFα can be produced independent of the NF-κB pathway. Two independent groups reported that TNFα could be triggered by zVAD and IAP antagonists through the RIPK1/AP1 and PKC/MAPKs/AP-1 pathways [8, 9]. The second means of production is NF-κB pathway-dependent. Another group reported that an IAP antagonist could activate NF-κB signaling and induce TNFα production to kill tumor cells [20, 21]. Depending on their response to IAP antagonists, cells can be grouped into three general classes :1) IAP antagonists induce autocrine TNFα production and massive cell death; 2) IAP antagonists induce autocrine TNFα production, but do not have a major effect on cell viability; 3) IAP antagonists have no effect on autocrine TNFα production or on cell death [20, 21, 36, 42, 43]. The ability of cells to produce TNFα is necessary but not sufficient for IAP antagonists to induce cell death as a single agent. Similar to IAP antagonists, which can induce TNFα production through activation of the non-canonical NF-κB pathway, NA activates the NF-κB pathway to enhance TNFα production and cell death. Two cellular events can result from cIAP degradation and include activation of the non-canonical NF-κB pathway with subsequently increased production of autocrine TNFα and caspase-8/RIPK1/FADD complex formation. Our results showed that NA activated the non-canonical NF-κB pathway stimulating the transcription of TNFα, but inhibited the canonical NF-κB pathway, which providing survival force to cells under stress. Specifically, the use of NA had the unintended, but fortuitous, effect of altering the functional state of TNFα from one that signals through the survival pathway to one that signals cell death. NA-induced TNFα autocrine production was only detected in NA-sensitive cell lines and not in NA-resistant lines. Why the TNFα-blocking antibody could not fully block NA-induced tumor cell death needs to be studied in the future. Although NA might influence other signaling pathways apart from non-canonical NF-κB, the fact that inhibition of non-canonical NF-κB signaling blocked TNFα production and promoted tumor cell survival in response to NA treatment suggests that non-canonical NF-κB signaling is the major pathway by which NA triggers TNFα production.

ROS has been implicated in TNFα-induced, RIPK-dependent necrosis [26, 39]. However, literatures suggest that cell-specific contexts might occur where the mitochondrial and necroptotic pathways bisect to mediate necrosis. Multiple studies have shown that mitochondrial ROS are critical in the execution of necroptosis in L929 cells [26, 44], whereas HT29 and U937 cells seem to lack this requirement [45]. Disparate results have been obtained in MEFs with ROS being both implicated in [46] and dispensable for [47] necroptosis. Although ROS production is not essential in all instances of necroptosis, increasing mitochondrial ROS production can act as second messengers in the signaling pathways leading to cell death. Treatment with NAC abrogated NA-induced ROS generation and cell death by necrosis, indicating that an imbalance in the oxidative stress response might be induced by NA. A previous study showed that RIPK3 physically interacts with and activates several metabolic enzymes to trigger ROS production [39]. Here, we observed that generation of ROS by NA also relies on RIPK3, but not RIPK1, and the mitochondrial complex I is the site for ROS generation. The relationship between RIPK3 and the metabolic process in NA-treated cells should be further investigated. Research results showed that the anti-necrotic effect of BHA reflects not only its ROS scavenging property, but also its ability to inhibit LOXs [40]. Here, we found that NA-induced ROS production is independent of PLA2 and LOXs. Taken together, our results show that mitochondrial complex I is the origin of ROS in NA-treated cells.

In summary, our study provides a mechanistic explanation for the observed ability of NA to stimulate cancer cell death and defines a novel biological role for RIPK/NF-κB in the regulation of cellular signaling. To further examine whether NA or other small-molecular natural compounds possess similar effects in other cell types, especially in apoptosis compromised human cancer cells, will be interesting to examine in the future.

## METHODS

### Cell lines and culture

The following cell lines were used: nasopharyngeal carcinoma cell lines (C666-1, HK-1, CNE1-LMP1), human amelanotic melanoma cell line (A375), human breast cancer cell line (MX-1), human gastric cancer cell line (AGS-EBV), and the mouse fibrosarcoma cell line (L929). All cells were cultured in RPMI-1640 medium (Gibco/BRL, Grand Island, NY) containing 10% fetal bovine serum (FBS), 100 units/ml penicillin and 100 mg/ml streptomycin. All cells were cultured in a humidified atmosphere with 5% CO_2_ at 37°C.

### Reagents

NA was isolated and identified from the mushroom *Albatrellus confluens* by State Key Laboratory of Phytochemistry and Plant Resources in West China, Kunming Institute of Botany, Chinese Academy of Sciences, Yunnan, (purity≥99%, HPLC analysis). Nec-1, zVAD-fmk, SP600125, NAI, and DMSO, were purchased from Sigma-Aldrich (St. Louis, MO). FBS and Lipofectamin were purchased from Invitrogen (Carlsbad, CA). TNFα blocking antibody was purchased from R&D Systems (Minneapolis, MN). Antibodies against NIK, p65 (Ser536) were purchased from Cell Signaling (Beverly, MA). The antibody against RIPK1 was purchased from BD Transduction Laboratories (Franklin Lakes, New Jersey). The antibody against β-actin was purchased from Sigma-Aldrich. Antibodies against TRAF2, TRAF3, TRAF6, cIAP1/2, His, IKKα, IKKβ, p65, p100/p52, IκBα, donkey anti-goat IgG-HRP, goat anti-rabbit IgG-HRP and goat anti-mouse IgG-HRP were purchased from Santa Cruz Biotechnology (Santa Cruz, CA).

### Plasmids

The following plasmids were used: His-Ub was gifted from Professor Qiao Wu (Xiamen University, China). His-UbK48-only and His-UbK63-only were generated in our lab. The NF-κB reporter plasmid pGL2-NF-κB-luc was gifted from Dr. Li Jianjian (National Institutes of Health, USA). The plasmid shRIPK3 was gifted from Professor Xiao-Dong Wang (National Institute of Biological Sciences, China). The pcdna3.1 hciap1 plasmid (Addgene 8311) and the FLAG-CIAP2 plasmid (Addgene 27973) were got from Addgene.

### Cell viability and flow cytometry assay

Cell viability was measured using a CellTiter-Glo Luminescent Cell Viability Assay kit (MTS) purchased from Promega Corp. (Madison, WI) and used according to the manufacturer's protocol. For flow cytometry analysis of apoptosis, cells were treated as indicated and harvested. Cells (1×10^6^ cells/ml) were resuspended in binding buffer and 0.5 ml of the suspension was transferred to a microfuge tube. After adding 5 μl Annexin V-FITC and 5 μl PI, cells were incubated at room temperature for 15 min in the dark. Apoptosis was analyzed by the Guava EasyCyte HT Sampling Flow Cytometer (Merck Millipore, Billerica, MA).

### Western blotting analysis

After harvesting and disrupting the cells, protein lysates were electrophoresed in SDS polyacrylamide gels and transferred to nitrocellulose membranes. Specific protein bands were visualized by an enhanced Western lightening plus-ECL kit (PIERCE, Rockford, IL). β-Actin was used as a loading control.

### Elisa analysis

Cells were plated onto 6-well plates and allowed to grow to approximately 80% confluence after 24 h. Media were then aspirated and the cells were washed 2 times with cold PBS. Fresh media (1 ml) were added and 100 ml aliquots were removed at each time point. Samples were kept at −80°C until ready for use. Elisa analysis was performed using a quantitative sandwich enzyme immunoassay from R&D Systems (TNFa Quanti-glo Chemiluminescent Elisa, QTA00B) according to the manufacturer's instructions.

### siRNA transfection

siRNA transfections were conducted in both 6-well and 24-well dish formats. Cells were cultured to 50% confluence after 24 h in antibiotic-free media. Approximately 48 h later, cells were collected and used for analysis. All siRNAs were purchased from Dharmacon (Thermo scientific, Pittsburgh PA). For all pooled siRNAs, Dharmacon's ON-TARGETplus siRNA-SMARTpool predesigned pools of four oligos were used and validated by Western blot. These included siRNAs for RIPK1 and IKKα.

RIPK1-1 target sequence, 5-CCACUAGUCUGACGGAUAA-3;

RIPK1-2 target sequence, 5-UGAAUGACGUCAACGCAAA-3;

RIPK1-3 target sequence, 5-GCACAAAUACGAACUUCAA-3;

RIPK1-4 target sequence, 5-GAUGAAAUCCAGUGACUUC-3.

IKKα-1 target sequence, 5-GGAGUUAGAGGCUGUGAUA-3;

IKKα-2 target sequence, 5-GCAGAUGACGUAUGGGAUA-3;

IKKα-3 target sequence, 5-GCGUGAAACUGGAAUAAAU-3;

IKKα-4 target sequence, 5-GCAAGUUGUUGGGCUGUAA-3.

### Real-time PCR

Total RNAs were isolated using Trizol (Invitrogen, Carlsbad, CA) according to the manufacturer's instructions. Total RNA (1 μg) was converted to cDNA using the Maxima First Strand cDNA Synthesis Kit for RT-qPCR (Thermo Scientific, Pittsburgh PA). To measure mRNA expression, quantitative real-time PCR was conducted using 2× SYBR green master mix on the ABI PRISM 7500 Sequence Detection qPCR machine (Applied Biosystems, Carlsbad, CA). The fold change in RNA was calculated using the comparative ΔΔCt method and normalizing to a *β-actin* control. The primers were synthesized by Sangon Biotech (Shanghai, China) and included the following:

*hTNFα* (forward: 5′-AGGACACCATGAGCACTGAAAGCA-3′;

reverse: 5′-TTGAGGGTTTGCTACAACATGGGC-3′);

*mTNFα* (forward: 5′-CTTCTCAT TCCTGCTTGTGG-3′;

reverse: 5′-ATGAGAGGGAGGCCATTTG-3′);

*β-actin* (forward: 5′-TTCCAGCCTTCCTTCCTGGG-3′;

reverse: 5′-TTGCGCTCAGGAGGAGCAAT-3′).

### Reactive oxygen species (ROS) detection

H2-DCFDA is widely used for ROS detection. H2-DCFDA is a stable, nonpolar compound that readily diffuses into cells and is hydrolyzed by nonspecific esterases to DCFH. This nonfluorescent molecule is further oxidized by ROS to form the fluorescent compound DCF. The cells were incubated with 10 μM H2-DCFDA at 37°C for 30 min, then harvested and the pellets suspended in 0.5 mL of PBS. ROS generation was measured as the increase in green fluorescence intensity using the FL1 channel on a Guava EasyCyte HT Sampling Flow Cytometer (Merck Millipore, Billerica, MA).

### Statistical analysis

Data are presented as means ± standard deviation. Statistical comparisons between different groups were determined by one-way ANOVA using the Statistics Package for Social Science (SPSS) software (version 16.0; SPSS, Chicago, IL, USA). A p value less than or equal to 0.05 was considered statistically significant.

## SUPPLEMENTARY MATERIAL, FIGURES, TABLES


