# Supplementary material for: Neoalbaconol induces cell death through necroptosis by regulating RIPK-dependent autocrine TNFα and ROS production
Source: Oncotarget. 2014 Dec 3;6(4):1995–2008. doi: 10.18632/oncotarget.3038 (PMC4385831; doi:10.18632/oncotarget.3038)
Supplement: Supplementary file 1 [file oncotarget-06-1995-s001.pdf]

Neoalbaconol induces cell death through necroptosis by regulating RIPK -dependent autocrine TNFα and ROS production

Supplementary Material

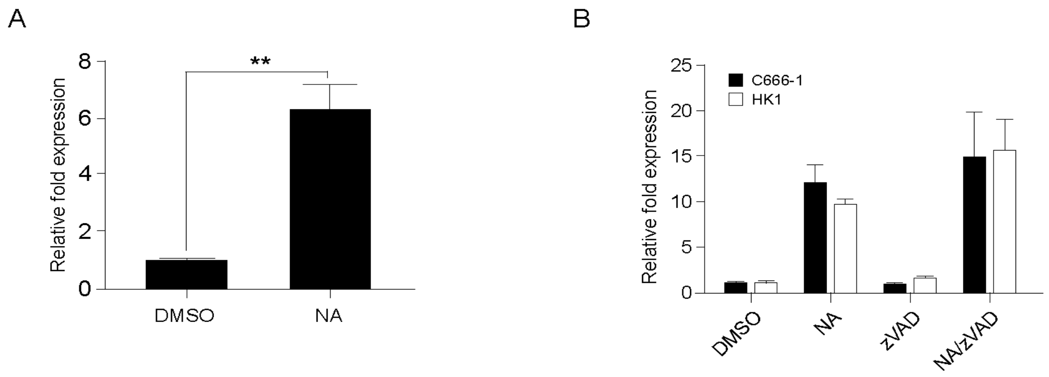

Supplementary Figure 1: NA, but not zVAD, promotes autocrine production of TNFα.

A. L929 cells were treated with NA (40 μM) for 8 h and the *TNFα* mRNA level was determined by quantitative-real time-PCR.

B. C666-1 and HK1 cells were pre-treated with zVAD (20 μM) for 30 min, and then treated or not treated with NA for 8 h. The *TNFα* mRNA level was determined by quantitative real time-PCR.

Each graphical representation indicates the means ± S.D. of at least three independent testing conditions. **p < 0.001.

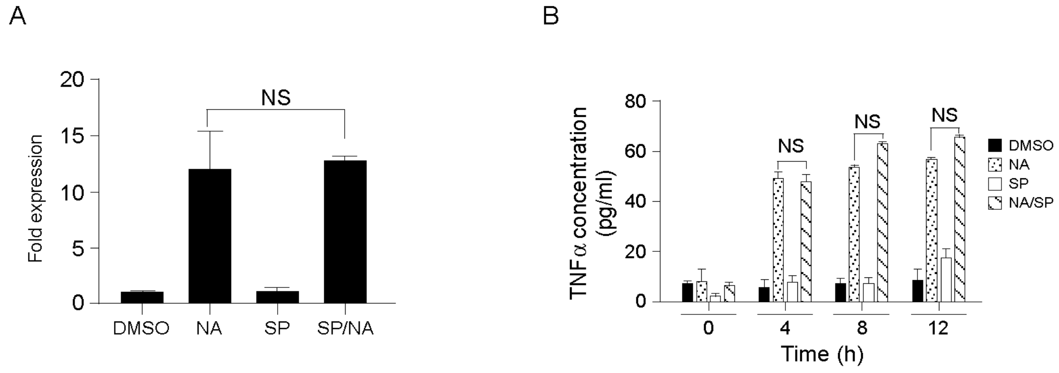

Supplementary Figure 2: JNKs are required for NA-induced TNFα production.

A. C666-1 cells were pre-treated with SP600125 (SP) for 1 h and then treated or not treated with 40 μM NA for 8 h. Relative levels of the TNFα transcript were determined and compared with β-actin and the fold change was calculated by comparing with DMSO-treated cells.

B. C666-1 cells were pre-treated with SP600125 (SP) for 1 h and then treated or not treated with 40 μM NA and harvested at the indicated time points. The presence of TNFα in conditioned cell culture media was measured by Elisa assay.

Each graphical representation indicates the means ± S.D. of at least three 3 independent testing conditions. ***p* < 0.001; ns, no significance.

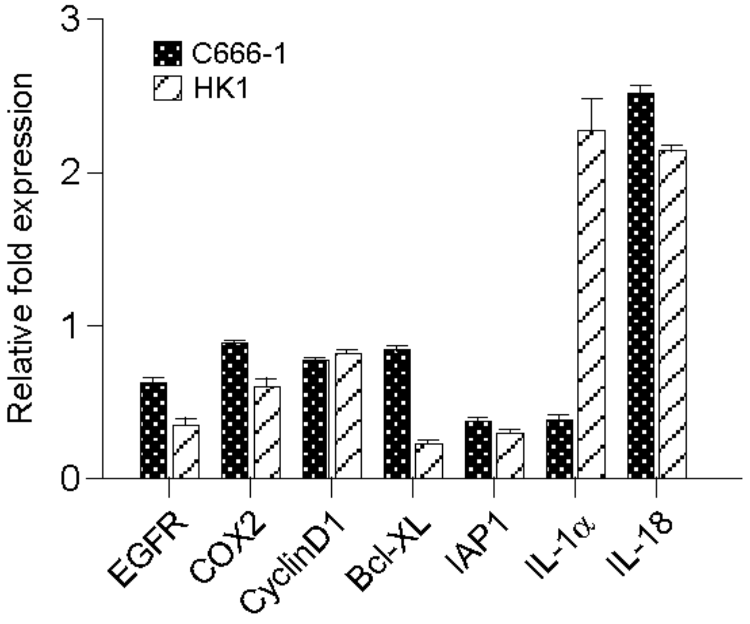

Supplementary Figure 3: The regulation of transcription factors by NA. The effect of NA-treatment on NF-κB-dependent anti-apoptotic, proliferative, and cytokine gene expression in C666-1 and HK1 cells was analyzed by quantitative-real time PCR. Each graphical representation indicates the means ± S.D. of at least three independent testing conditions.

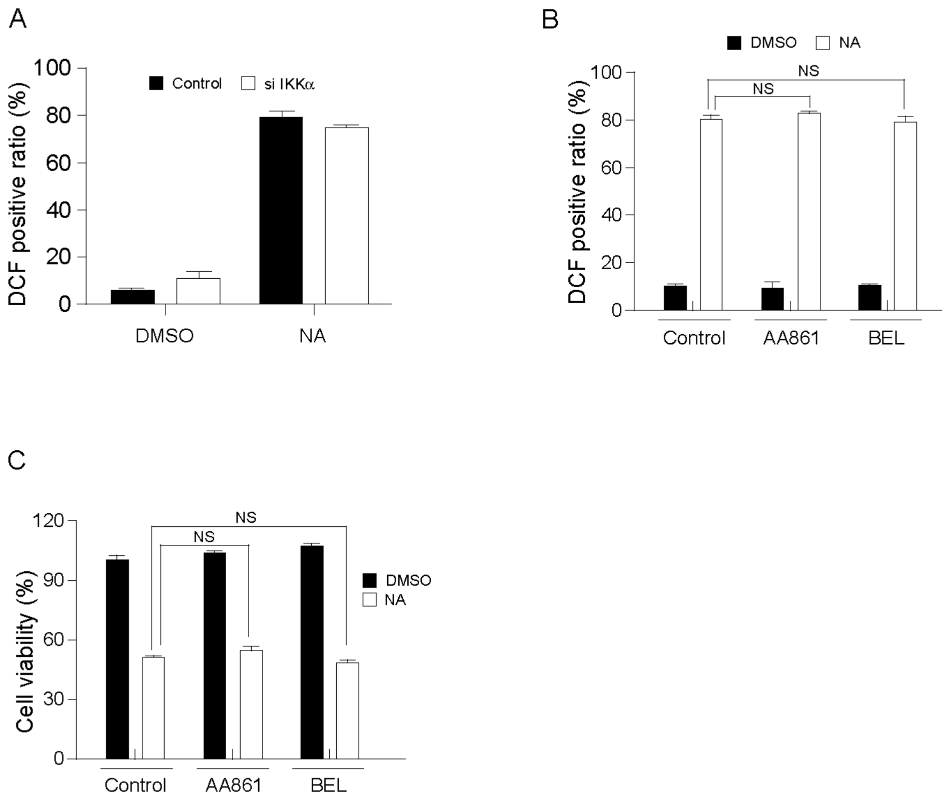

Supplementary Figure 4: NA leads to mitochondrial dysfunction in cancer cells.

A. C666-1 cells were transfected with siRNA mock or siRNA targeting IKKα for 48 h, and then treated or not treated with 40 μM NA. ROS were measured using the dye DCF at 12 h after stimulation.

B. PLA2 and LOX do not participate in NA-induced cell death. C666-1 cells were pre-treated for 30 min with medium, 50 μM AA861 or 30 μM BEL, and then treated with either 10 mM LiCl or 40 μM NA + 10 mM LiCl. ROS production and cell death were determined by FCM and MTS assay.

Each graphical representation indicates the means ± S.D. of at least 3 independent testing conditions. *p < 0.05. **p < 0.001; ns, no significance.
